# Elaboration and synthesis of the whitlockite phase using limestone dust: *in vitro* bioactivity for biomedical applications

**DOI:** 10.1039/d5ra05840f

**Published:** 2025-09-24

**Authors:** H. Agourrame, N. Khachani, A. Diouri, L. Rghioui, A. Zarrouk

**Affiliations:** a Laboratoire de Chimie Appliquée des Matériaux, Centre des Sciences des Matériaux, Faculty of Science, Mohammed V University in Rabat Avenue Ibn Battouta BP 1014 Rabat Morocco hind.agourrame@gmail.com +212615050686; b Laboratoire de Spectroscopie, Modélisation Moléculaire, Matériaux, Nanomatériaux, Eau et Environnement, Department of Chemistry, Faculty of Science, Mohammed V University in Rabat Avenue Ibn Battouta BP 1014 Rabat Morocco; c Laboratory of Nanotechnology, Materials and Environment, Faculty of Sciences, Mohammed V University in Rabat Av. Ibn Battouta, BP 1014 RP M-10000 Rabat Morocco azarrouk@gmail.com +00212665201397

## Abstract

Calcium silicate-based bioceramics have attracted attention in biomedical applications due to their calcium and silicon contents, which are essential for bone health. Whitlockite (WH), the second most abundant inorganic component of bones after hydroxyapatite (HAP), constitutes 20–35% of bones by weight and exhibits excellent biological properties, making it particularly attractive for tissue engineering. It is therefore essential to synthesize the WH phase using cost-effective and eco-friendly by-products. This study aims to synthesize the whitlockite phase using dicalcium silicate (larnite, Ca_2_SiO_4_). Dicalcium silicate was synthesized from a mixture of limestone dust (LD), a by-product consisting essentially of calcium carbonate (CaCO_3_), and soda lime glass powders, as a source of silicon dioxide (SiO_2_). Additionally, the surface reactivity and bioactivity of the composite sample were evaluated *in vitro* by immersing it in artificial saliva (SA) and in a simulated body fluid (SBF) for time periods ranging from 1 hour to 5 hours. The mineralogical and microstructural properties of the samples were characterized using XRD, FTIR and SEM analysis techniques. The characterization of the obtained powders indicated that the whitlockite phase synthesized through a co-precipitation method exhibited good bioactivity both in artificial saliva (SA) and simulated body fluid (SBF). Moreover, the analysis revealed the development of a hydration phase of rosenhahnite (Ca_3_Si_3_O_10_H_2_) and the formation of a hydroxyapatite (Ca_5_(PO_4_)_3_(OH)) phase within 1 hour of immersion in both bioactive media. The morphology of the samples was analyzed using SEM, which showed significant grain growth and consolidation after 1 hour of immersion in artificial saliva and simulated body fluid. After 5 hours, the grains appeared to be well connected with each other, indicating good consolidation.

## Abstract 2

Calcium silicate bioceramics are promising for biomedical use owing to Ca/Si being essential for bone health. Whitlockite, the second most abundant bone mineral after hydroxyapatite, shows excellent bioactivity for tissue engineering.

## Graphical abstract

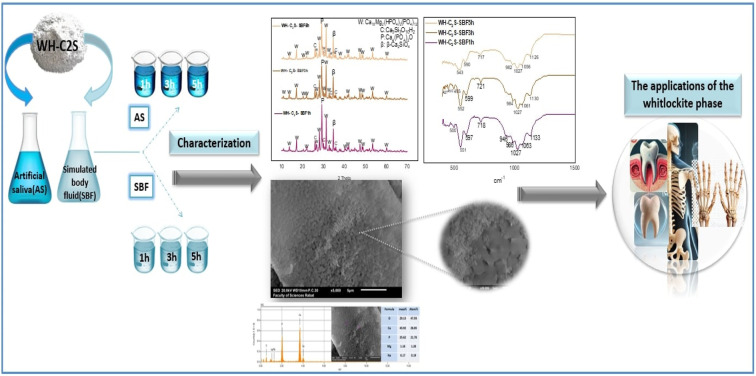

## 1. Introduction

Whitlockite (WH) is the second-most major bone mineral, which is attracting interest for its application in bone regeneration as it promotes growth and supports bone tissue development from stem cells.^1^ Magnesium whitlockite (WH: Ca_18_Mg_2_(HPO_4_)_2_(PO_4_)_12_) is an orthophosphate compound that incorporates both calcium (Ca^2+^) and magnesium cations (Mg^2+^) and phosphate anions (HPO_4_^2−^); it is formed by partially exchanging magnesium ions with calcium ions in the crystal structure of calcium orthophosphate.^2^ Whitlockite (WH) is a biomaterial present in bone and dentin,^3^ and it has also been identified in salivary stones with apatites.^4^ Although rare in nature, it constitutes 20–35% of human bones, after hydroxyapatite, especially in bones subjected to high loads.^5^ Dentin contains 26–58% of whitlockite, and Mg-whitlockite is a biomineral commonly found in mineralized dental tissues.^1^ Additionally, whitlockite is recognized as the fastest-dissolving mineral phase of the bone tissue.^2^ In particular, compared with hydroxyapatite (HAP) under acidic conditions, whitlockite (WH) is a relatively stable material.^6^ Furthermore, it has been shown to be more effective than hydroxyapatite in stimulating osteogenesis,^7^ and it is a promising bioceramic for bone replacement despite its complex structure.^8,9^ It has been shown in a recent study that magnesium ions (Mg^2+^), a well-known inhibitor of HAP growth, play an essential role in the formation of WH.^10^ Several methods have been explored to synthesize the whitlockite phase, including hydrothermal, sol–gel, wet, and solid-state routes, with varying results obtained in terms of purity and ionic composition.^17^ Nevertheless, these methods frequently encounter limitations, such as the need for high temperatures,^11^ low thermodynamic stability of WH,^12^ or a narrow pH range; for example, the sol–gel route requires heat treatment at temperatures up to 1100 °C.^13^ Similarly, synthesis under hydrothermal conditions requires a high temperature to achieve the desired purity.^14^ Furthermore, attempts to incorporate magnesium into other calcium phases have only resulted in multiphase mixtures.^15^ In wet synthesis, achieving simultaneous control of parameters remains a challenge.^12^ Thus, despite numerous studies, obtaining a WH with a controlled composition remains complex.^12^ Previous studies have shown that Ca–Si–Mg-containing bioceramics exhibit high bioactivity, making CaO–MgO–SiO_2_ systems promising third-generation bone graft substitutes for tissue engineering.^16^ These ceramics not only exhibit superior biological performance but also enhance bone repair and regeneration capabilities upon the addition of bone progenitor cells and growth factors.^17^ For example, clinoenstatite (MgSiO_3_) is a silicate phase with remarkable mechanical, chemical and biological properties, making it promising for applications in tissue engineering.^18^ Furthermore, calcium silicates such as wollastonite and dicalcium silicate efficiently release calcium (Ca) and silicon (Si) ions, confirming their application potential for bone regeneration and replacement, as demonstrated by *in vitro* and *in vivo* studies.^19^ In addition, multiple studies have substantiated the bioactivity of Ca_2_SiO_4_ powder.^20^ Dicalcium silicate, particularly in its β-Ca_2_SiO_4_ (belite) form, is attracting considerable interest due to its diverse applications, including in cement, ceramics, pharmaceuticals, and biomaterials.^21^ The β-dicalcium silicate (β-C_2_S) phase is generally synthesized by calcining a calcium carbonate/silica mixture at approximately 1000 °C.^22^ Heat treatments between 600 and 1000 °C produce this phase, confirmed by X-ray diffraction and microscopy.^23^ In a water vapor atmosphere, the formation of β-C_2_S can occur at temperatures as low as 650 °C, while in dry air, it requires at least 800 °C.^24^ Sintering at 1050 °C allows for the complete stabilization of the C_2_S phase.^25^ The addition of zinc and sequential treatments with rapid cooling improve the synthesis.^26^ Grinding with ethanol increases the reactivity of the precursors and optimizes the process.^27^ This form (belite) has been shown to promote the rapid formation of an apatite layer on its surface when immersed in a simulated body fluid (SBF).^28^ Similarly, it induces the precipitation of hydroxyapatite after immersion in artificial saliva.^29^ This bioactivity is linked to the release of Ca^2+^ and SiO_4_^4−^ ions, which induce the precipitation of an apatite layer.^30^ This formation is observed even in environments not rich in calcium and silicon or phosphate.^31^ The mechanism is based on the adsorption of phosphate ions, followed by their crystallization into apatite.^32^ Several studies have shown that the formation of hydroxyapatite (HAP) can be detected after 5 to 6 hours of immersion.^33^ Other studies indicate that an initial layer of HAP begins to develop after 10 hours, although its complete maturation requires more time.^34^ These observations are explained by the intense ionic exchanges that occur during the first hours in the simulated body fluid.^35^ Furthermore, microscopic analyses confirm the presence of HAP after only 5 hours of immersion.^36^ In this context, a recent study has shown that mussel shells and soda-lime glass powders can be used to synthesize dicalcium silicate by a solid–solid reaction.^37^ This material, as well as limestone-silica mixtures, exhibits interesting bioactivity in an SBF solution, making it promising for implant applications.^38^ Furthermore, β-wollastonite obtained from a mixture of limestone and rice straw ash reveals bioactive properties suitable for implant applications.^39^ The use of soda-lime-silica glass waste promotes the formation of bioactive, recyclable glass-ceramics suitable for dental restoration.^40,41^ In contrast to conventional methods that require high processing temperatures or high-purity reagents, our approach relies on the use of industrial by-products, making it more cost-effective and environmentally friendly. This study investigates the synthesis of whitlockite-dicalcium silicate (WH-C_2_S) powder from by-products such as limestone dust and soda-lime glass powder *via* a solid-state reaction, followed by a co-precipitation process. The bioactivity of the WH-C_2_S powder was examined by immersing it in artificial saliva (SA) and simulated bodily fluid (SBF) at various time intervals ranging from 1 hour to 5 hours. The resulting products were analyzed before and after immersion to assess the changes in their bioactivity. The mineralogical development of the synthesized phases was monitored using X-ray diffraction (XRD) patterns, recorded using Cu Kα radiation with a wavelength of 1.5406 Å, and infrared spectroscopy (FT-IR), and the microstructure of the samples was examined by scanning electron microscopy (SEM).

## 2. Experimental

### 2.1. Starting materials

Dicalcium silicate (C_2_S) was synthesized *via* a solid-state reaction from a reactant mixture of limestone dust (LD), primarily composed of calcium carbonate (CaCO_3_), and soda-lime glass powders, serving as a source of SiO_2_, maintaining a Ca/Si ratio of 2. For this study, the particle size used was less than 40 μm. The synthesis entailed heating finely ground mixtures within a temperature range of 100 °C to 1000 °C, followed by rapid air cooling. The thermal treatments were intermittently halted for grinding, with ethanol used to augment product reactivity, followed by rapid cooling in air. The obtained powder (C_2_S) was dissolved separately in deionized water. Subsequently, 1 M phosphoric acid solution (H_3_PO_4_, 85%) was added, with a Ca/P ratio of 1.42. The resulting mixture was stirred for 4 hours at a temperature of 75 °C. Then, the precipitates were subjected to vacuum filtration, washed with distilled water, and dried at 80 °C. Finally, the powder obtained was subjected to calcination at a temperature of 700 °C for 4 hours. The prepared samples were respectively denoted as WH-C_2_S. To prepare WH-C_2_S pastes, distilled water was used as the liquid phase to mix the powders and enable the setting reactions. The WH-C_2_S powder was meticulously combined with distilled water in a liquid-to-powder (L/P) ratio of 0.6 mL g^−1^, yielding a homogeneous paste for immersion in diverse settings, including artificial saliva and simulated bodily fluid.

The simulated body fluid (SBF) and artificial saliva (SA) were prepared according to the chemical composition of human body fluids and saliva, with ion concentrations identical to those of the inorganic components of blood plasma and natural saliva. The aim of this approach is to assess the *in vitro* bioactivity of the whitlockite-dicalcium silicate (WH-C_2_S) bioceramic and to compare it with the *in vivo* bioactivity.

### 2.2. *In vitro* bioactivity in artificial saliva

WH-C_2_S powder was immersed in artificial saliva (SA) for 1, 3, and 5 hours, yielding WH-C_2_S-SA1h, WH-C_2_S-SA3h, and WH-C_2_S-SA5h, respectively. As shown in Table 1,^23,37^ the AS solution corresponds to the SAGF medium. 1 M hydrochloric acid and ultra-pure water (resistivity of 18.2 MΩ cm at 25 °C; a total organic carbon (TOC) content of less than 5 ppb; and ion and particle contents of less than 1 ppb and 1 particle per mL, respectively) with a pH of 6.8, which is comparable to that of natural saliva, were employed to regulate the pH level. To evaluate the bioactivity of the samples, they were contained in polyethylene bottles with 10 mL of saliva and incubated at a stable temperature of 37 ± 2 °C.^42^

**Table 1:** Constitution of the artificial saliva solutions employed in the study (SAGF medium)^37^

Substances	NaCl	KCl	CaCl_2_·2H_2_O	KH_2_PO_4_	Urea	NH_4_Cl	NaHCO_3_	Na_2_SO_4_ 10H_2_O	KSCN
Conc. (g L^−1^)	0.125	0.963	0.227	0.654	0.200	0.178	0.630	0.763	0.189
Conc. (mmol L^−1^)	2.14	12.92	1.54	4.81	3.33	3.33	7.50	2.37	1.94

### 2.3. *In vitro* bioactivity in simulated body fluid (SBF)

The bioactivity assessment of the WH-C_2_S powder was carried out utilizing a simulated body fluid (SBF) at a temperature of 37 ± 2 °C.^43^ The powders were immersed in SBF for various durations: 1, 3, and 5 hours. The powders were named WH-C_2_S-SBF1h, WH-C_2_S-SBF3h and WH-C_2_S-SBF5h, respectively. The SBF solution in this case carefully represents an ion-concentration medium similar to human blood plasma (as shown in Table 2). The SBF solution was made up of NaCl, NaHCO_3_, KCl, MgCl_2_, 1 M HCl, CaCl_2_·6H_2_O, Na_2_HPO_4_ and Na_2_SO_4_, corresponding to the Sigma-Aldrich in St. Louis, MO, USA. The buffer pH based on the HCO_3_^−^/CO_2_ (or H_2_CO_3_) pair was carefully adjusted to 7.4 with a solution of HCl (1 M).

**Table 2:** Simulated body fluid (SBF) composition

Ion	Concentration/mol m^−3^	
	SBF	Human blood plasma
Na^+^	142.0	142.0
K ^+^	5.0	5.0
Mg^2+^	1.5	1.5
Ca^2+^	2.5	2.5
Cl^−^	147.8	103.0
HCO^3−^	4.2	27.0
HPO_4_^2−^	1.0	1.0
SO_4_^2−^	0.5	0.5

The pH of each solution (for pH measurement, each point was measured three times (*n* = 3)) was regularly monitored before any sample was withdrawn (Table 3), and subsequently, the powders were soaked in acetone solution for at least 24 hours to halt the hydration reaction.^38^ Following air drying, the samples underwent characterization by FT-IR, SEM, and XRD investigations to observe the bioactivity of the bioceramic phases during the immersion period. The abbreviations utilized in this study are listed in Table 4.

**Table 3:** pH evolution during immersion in artificial saliva (SA) and in simulated body fluid as a function of the immersion time

Immersion period (h)	1	3	5
pH (artificial saliva)	5.8	6.9	6.9
pH (simulated body fluid)	5.8	6.7	7.2

**Table 4:** List of abbreviations and their meaning in the present paper

Meaning	Abbreviation
Limestone dust	LD
Simulated body fluid	SBF
Artificial saliva	SA
Dicalcium silicate/larnite	C_2_S
Whitlockite	WH
Hydroxyapatite	HAP
After co-precipitation of dicalcium silicate (C_2_S)	WH-C_2_S
After immersing the powders (WH-C_2_S) in SA for 1, 3, and 5 hours	WH-C_2_S-SA1h, WH-C_2_S-SA3h, WH-C_2_S-SA5h
After immersing the powders (WH-C_2_S) in SBF for 1, 3, and 5 hours	WH-C_2_S-SBF1h, WH-C_2_S-SBF3h, WH-C_2_S-SBF5h

### 2.4. X-ray fluorescencet table of contents entry

Table 5 indicates the chemical composition of the limestone dust and soda-lime glass powders obtained by XRF analysis in this study. Analysis reveals that limestone dust is composed of 52.09% calcium oxide (CaO) by mass, 3.279% by mass of magnesium oxide (MgO), as well as small proportions of aluminium oxide (Al_2_O_3_), silicon dioxide (SiO_2_) and other oxides in minor quantities. Mass loss is measured by taking the mass of the sample before and after calcination at 1000 °C. The mass loss observed for limestone dust (40.34% by weight) is due to the decomposition of calcium carbonate. For soda-lime glass powder (Table 5), the major element is silicon dioxide (SiO_2_) with a mass percentage of 72.75%, followed by calcium oxide (7.618%) and then magnesium oxide (3.091%). We also found elements in trace form, such as aluminum oxide (Al_2_O_3_) and potassium oxide.

**Table 5:** Chemical composition of limestone dust and soda-lime glass powder (in % by mass)

Elements	Limestone dust	Glass powder
CaO	52.09	8.61
MgO	3.27	4.09
Na_2_O	0.069	2.45
SiO_2_	1.92	78.75
SO_3_	0.08	0.27
Al_2_O_3_	1.21	1.48
SrO	0.02	—
MnO	0.08	—
Fe_2_O_3_	0.68	0.43
Tm_2_O_3_	0.02	—
P_2_O_5_	0.01	—
K_2_O	0.10	0.43
NiO	0.01	—
Rb_2_O	0.002	—
Cl	—	0.06
TiO_2_	—	0.05
*PF	40.34	10.15
Total	100	100

## 3. Results and discussion

### 3.1. X-ray diffraction

**Fig. 1:**
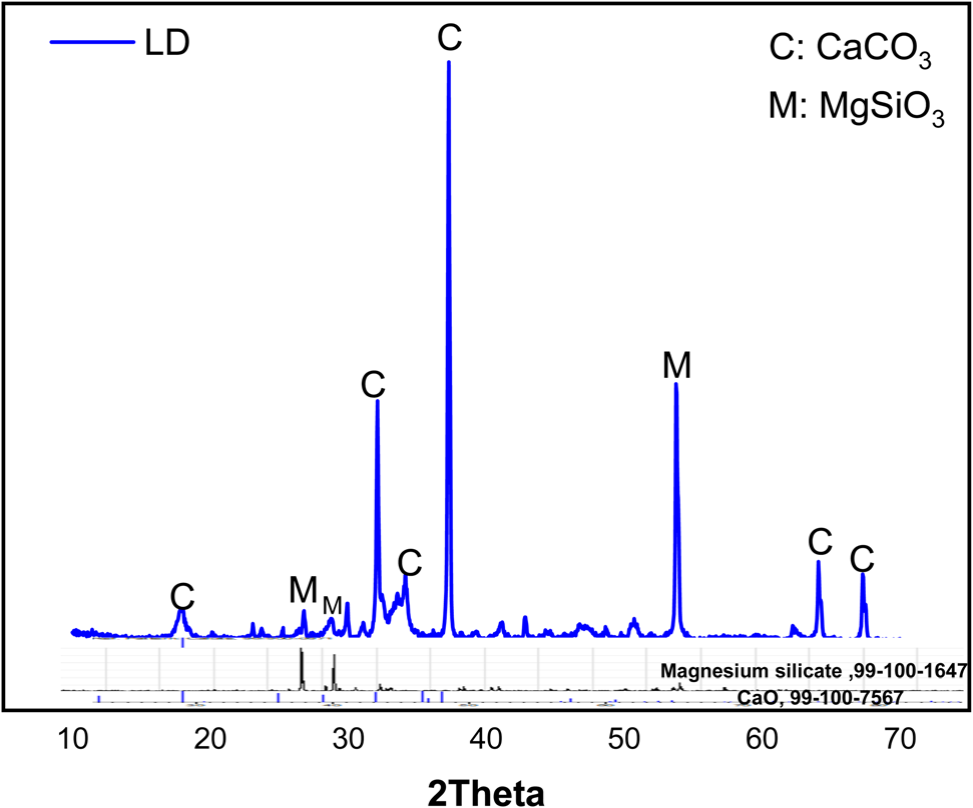
X-ray diffractogram of limestone dust (LD).

The mineralogical composition of limestone dust is obtained by XRD (X-ray diffraction). This composition is represented by the X-ray diffractogram in Fig. 1. The analysis shows that the main crystalline phases are calcium oxide (CaO, PDF 99-100-7567) and magnesium silicate (MgSiO_3_, PDF 99-100-1647).

**Fig. 2:**
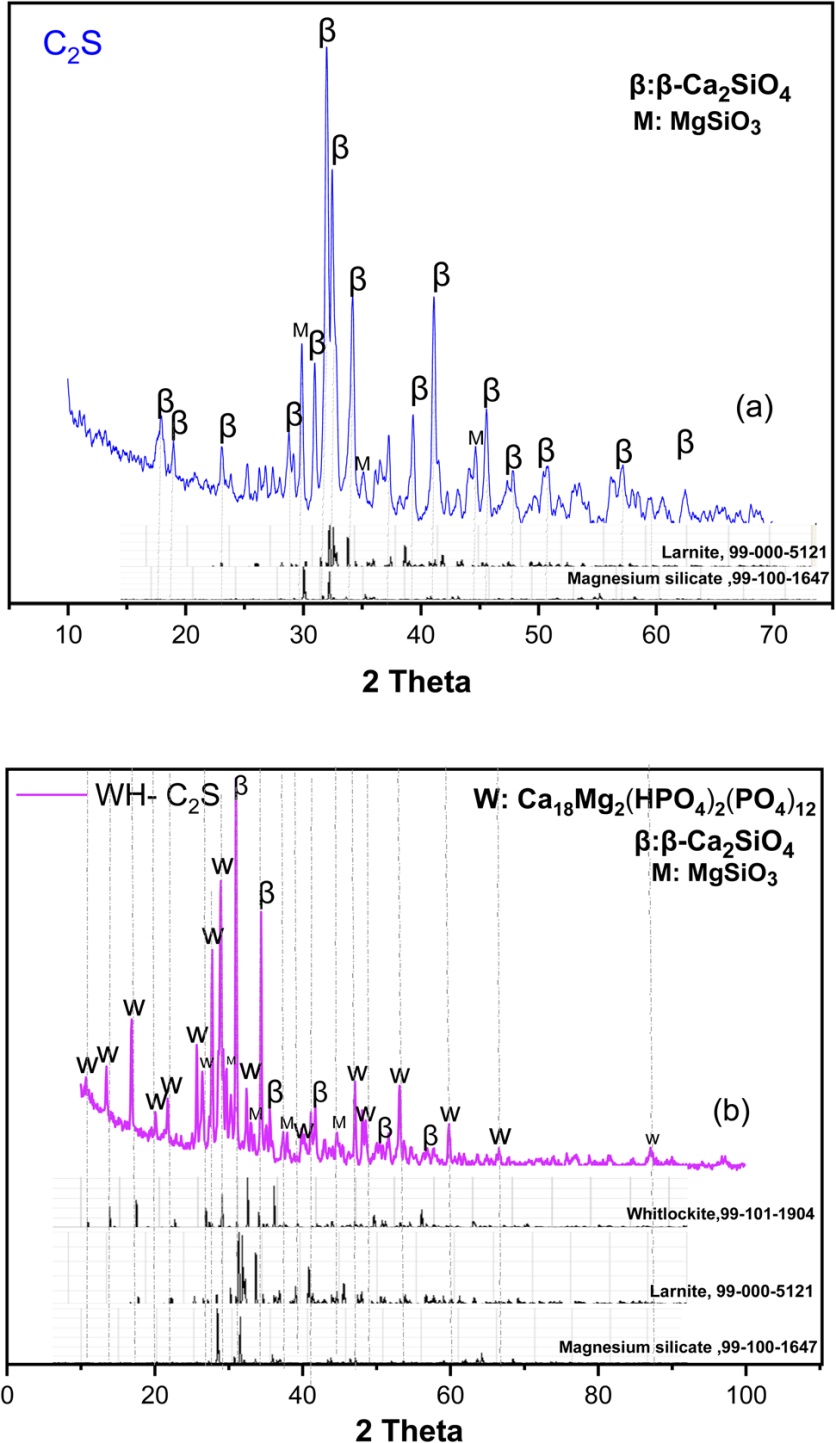
X-ray diffractogram of the C_2_S (a) and WH-C_2_S powders (b).

Fig. 2 shows the X-ray diffractograms of the synthesized C_2_S and WH-C_2_S powders at 1000 °C. The XRD analysis (Fig. 2(a)) confirms the presence of β polymorphs in the monoclinic crystal system, with the larnite phase (β-Ca_2_SiO_4_, PDF 99-000-5121) exhibiting very intense peaks, clearly indicating its predominance. Peaks corresponding to magnesium silicate (MgSiO_3_, PDF 99-100-1647) are also observed in the diffractogram. After co-precipitation (Fig. 2(b)), a diminution in the peak intensities of the β-Ca_2_SiO_4_ and MgSiO_3_ phases is observed, with the decrease of the β-Ca_2_SiO_4_ peak mainly attributed to its dissolution through hydration. This diminution of β-Ca_2_SiO_4_ phase is accompanied by the appearance of characteristic high-intensity peaks of a new phase, whitlockite (WH: Ca_18_Mg_2_(HPO_4_)_2_(PO_4_)_12_ PDF 99-101-1904), indicating its predominance.

**Fig. 3:**
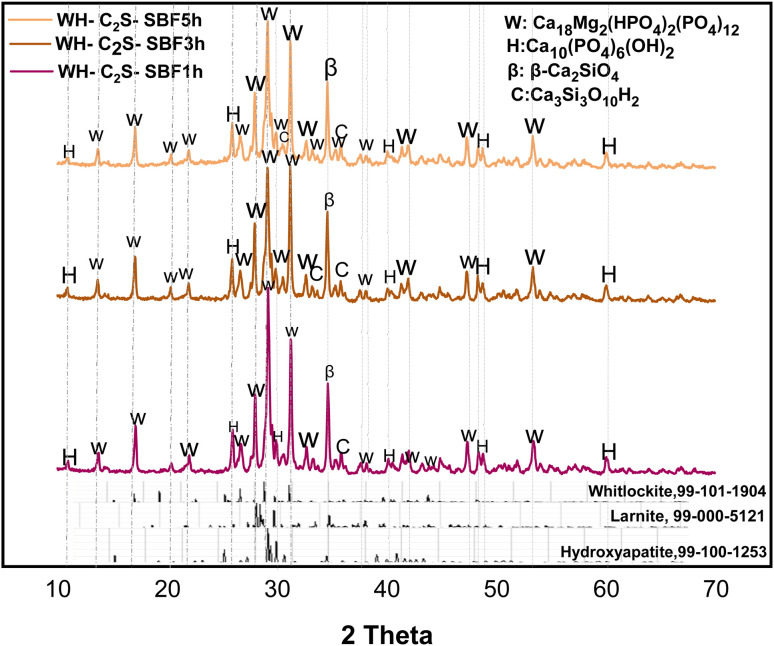
XRD diffractograms of WH-C_2_S-SA1h, WH-C_2_S-SA3h, and WH-C_2_S-SA5h after immersion in artificial saliva (SA).

**Fig. 4:**
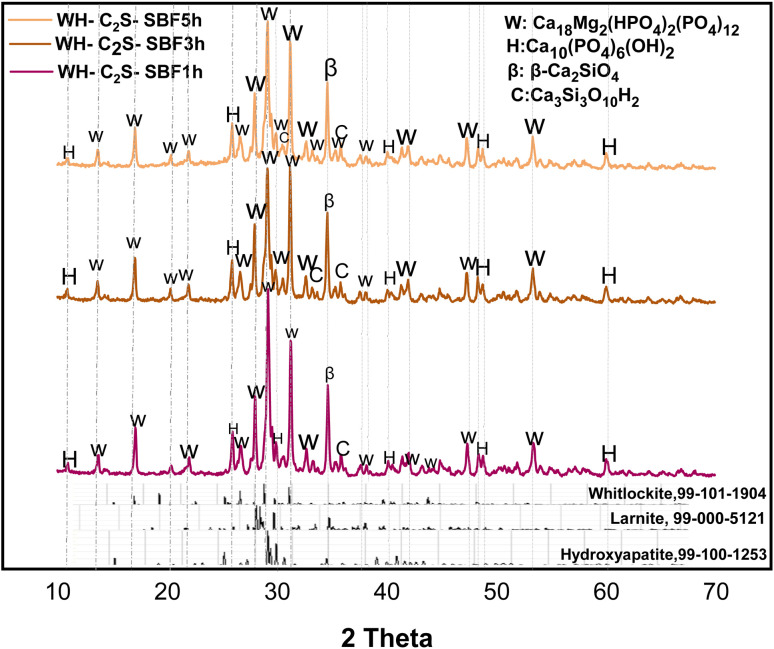
XRD diffractograms of WH-C_2_S-SBF1h, WH-C_2_S-SBF3h, and WH-C_2_S-SBF5h after immersion in simulated body fluid (SBF).

After immersing the powders and air drying, each sample was subjected to X-ray diffraction (XRD) analysis. X-ray diffraction patterns of the samples after 1, 3 and 5 hours of immersion in saliva and simulated body fluid (Fig. 3 and 4) show the disappearance of the characteristic peaks of the MgSiO_3_ phase, as well as the reduction in the peak intensities of the larnite (β-Ca_2_SiO_4_) phase. At the same time, new phases appear. This is explained by the reaction established between the prepared bioactive medium (artificial saliva or simulated body fluid) and the WH-C_2_S powder during its immersion. The reaction promotes the hydration and dissolution of the powder, leading to the formation of rosenhahnite hydration (Ca_3_Si_3_O_10_H_2_, PDF 99-100-0578) and hydroxyapatite ((Ca_5_(PO_4_)_3_(OH), PDF 99-100-1253) phases after 1 hour of immersion in SA and SBF, respectively. We also observe the growth of the characteristic peaks of the hydroxyapatite phase with immersion in both bioactive media as a function of time. A significant increase in the intensity of the peaks associated with the whitlockite phase is noted with time intervals ranging from 1 hour to 5 hours, in the case of powders immersed in SBF. In addition, after immersion in the simulated body fluid (SBF) and artificial saliva (SA), the samples increased the pH of both solutions. The pH increase is accompanied by a decrease in the intensity of the characteristic peaks of the larnite (β-Ca_2_SiO_4_) and MgSiO_3_ phases, indicating a significant dissolution of the phases. Additionally, the characteristic peaks of the Whitlockite phase in the simulated body fluid are more intense compared to those for the artificial saliva medium.

### 3.2. Spectroscopy FTIR

**Fig. 5:**
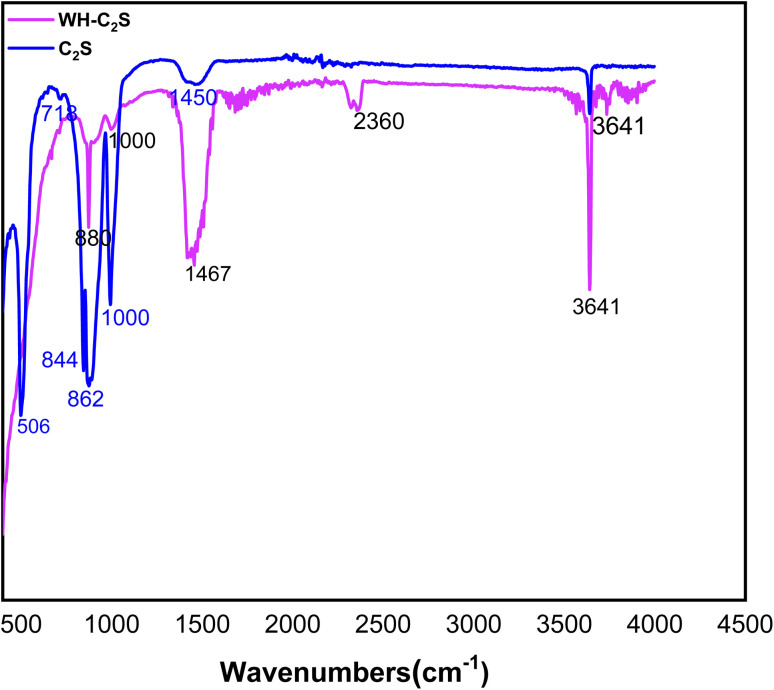
FT-IR spectra of the C_2_S and WH-C_2_S powders.

The FTIR spectra of the C_2_S and WH-C_2_S powders are shown in Fig. 5. The absorption bands detected between 800 and 1000 cm^−1^ correspond to the symmetric and asymmetric vibrations of the Si–O bonds present in the dicalcium silicate structure,^44^ as well as to the P–O stretching vibrations of the PO_4_ group of the whitlockite phase.^45^ The bands located between 502 and 718 cm^−1^ are attributed to the deformation vibrations of the O–P–O^46^ and O–Si–O bonds.^47^ The absorption bands between 1431 cm^−1^ and 1460 cm^−1^ are related to the stretching vibrations of the CO_3_^2−^ group, indicating the presence of CaCO_3_. Finally, the band at 3645 cm^−1^ corresponds to the O–H stretching of the water molecules absorbed by our compound, which is hygroscopic.^48^ In addition, a very intense OH absorption band is observed around 3600 cm^−1^, attributed to the presence of incorporated water.^49^

**Fig. 6:**
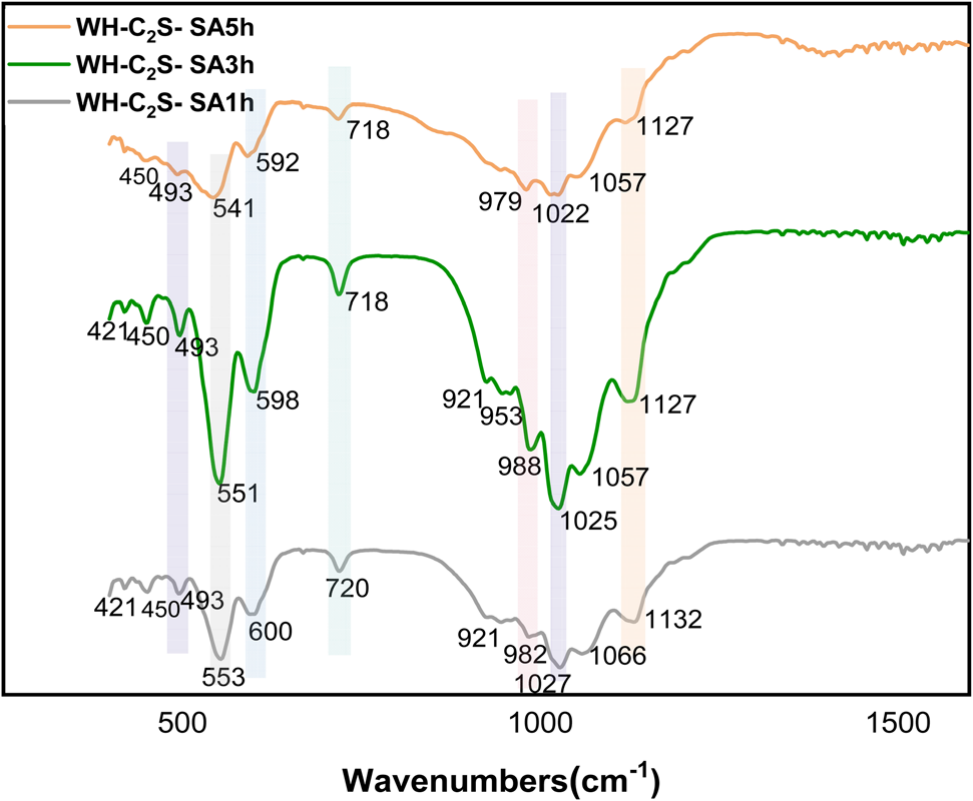
FT-IR spectra of the WH-C_2_S-SA1h, WH-C_2_S-SA3h, and WH-C_2_S-SA5h powders after immersion in artificial saliva (SA).

**Fig. 7:**
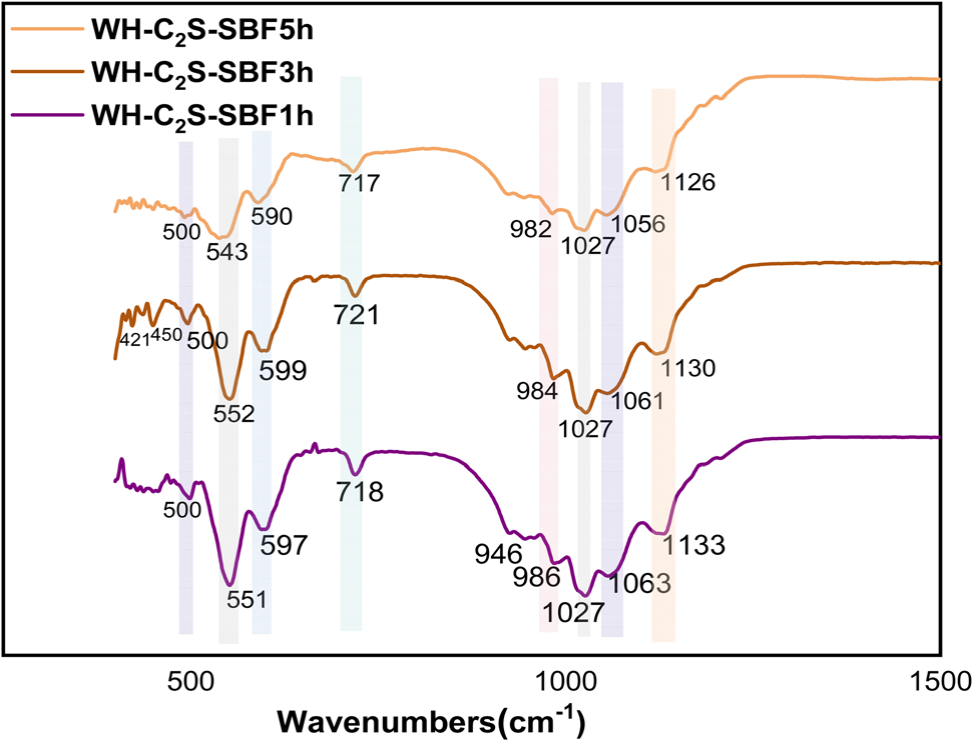
FT-IR spectra of the WH-C_2_S-SBF1h, WH-C_2_S-SBF3h, and WH-C_2_S-SBF5h powders after immersion in simulated body fluid (SBF).

The FTIR spectra of the WH-C_2_S powders after 1 hour to 5 hours of immersion in artificial saliva and in simulated body fluid are shown in Fig. 6 and 7, respectively. The spectra of the powder samples after immersion in artificial saliva and simulated body fluid reveal pronounced similarities, with the exception of a small difference for the samples immersed for 5 hours, whose peak intensities decreased with increasing time in comparison with the other spectra. The absorption bands detected between 800 and 1000 cm^−1^ correspond to the symmetric and asymmetric vibrations of the Si–O bonds in the dicalcium silicate structure, while the bands between 400 and 500 cm^−1^ are attributed to the O–Si–O vibrations.^50^ Furthermore, the absorption peaks detected around 920 cm^−1^ (ref. 51) and 922 cm^−1^,^52^ as well as those located between 1000 and 1140 cm^−1^, reveal the presence of the P–O bonds of the HPO_4_^2−^ group.^53^ Finally, the absorption bands located between 555 and 603 cm^−1^ are attributed to the deformation vibrations of the O–P–O bonds.^54^ The band observed around 721 cm^−1^ can be attributed to the valence vibration of the Si–O–Si bridge of rosenhahnite (Ca_3_Si_3_O_10_H_2_).^55^ The band at 1636 cm^−1^ and the bands at 1413 and 2362 cm^−1^ are attributed to the presence of CO_3_^2−^ group.^56^ Finally, the band around 3400 cm^−1^ corresponds to the O–H stretching of absorbed water.^48^

#### 3.3. Scanning electron microscopy (SEM)

**Fig. 8:**
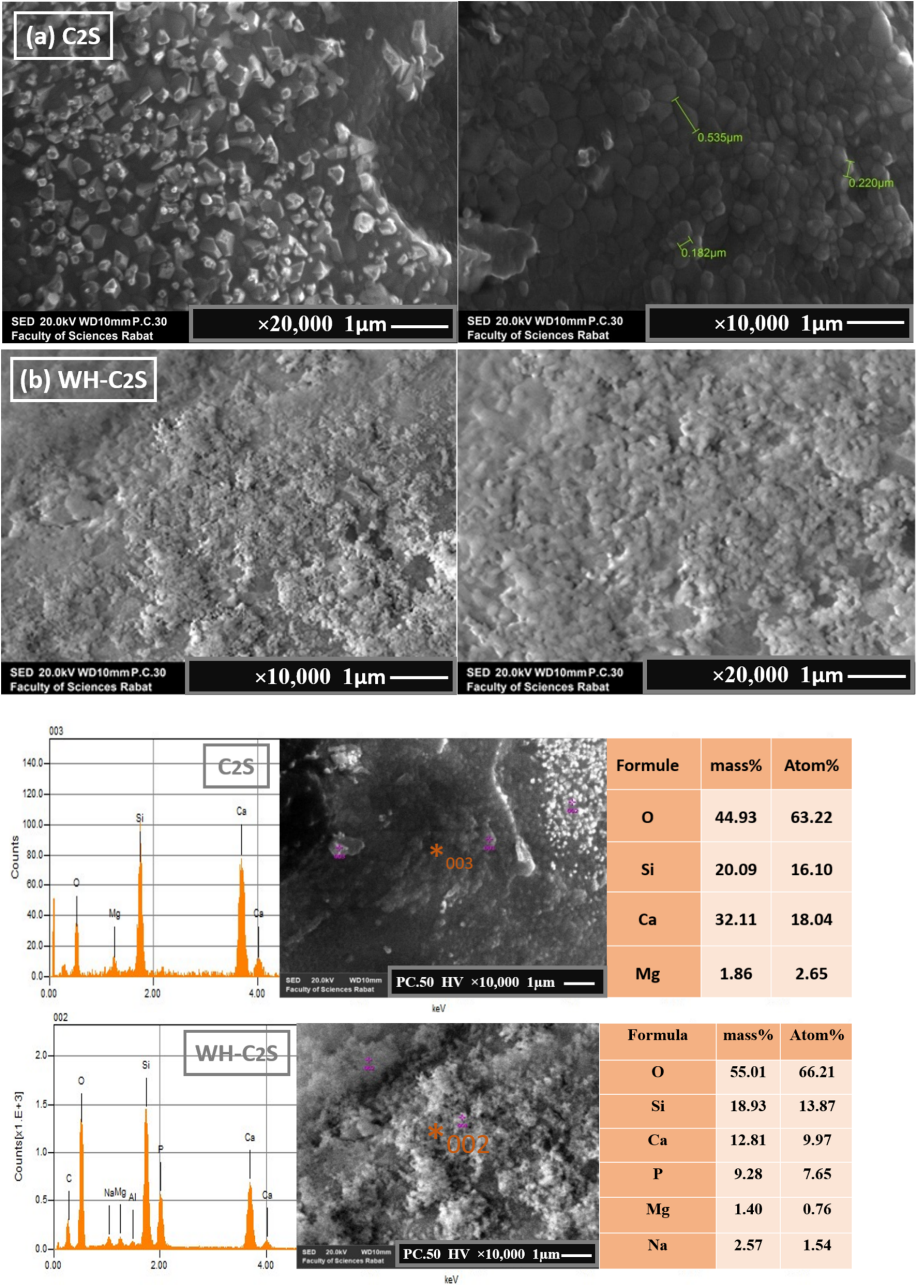
SEM images and EDS of C_2_S (a) and WH-C_2_S (b) samples after immersion in artificial saliva (SA).

The micrographs obtained by scanning electron microscopy (SEM) analysis of the C_2_S and WH-C_2_S samples are shown in Fig. 8(a). We determined the average thickness of the formed particles (1 μm ± 0.3 μm) from five analyzed areas. The morphology of the C_2_S sample reveals near-spherical particles with an average size of 0.18 to 0.53 μm, attributed to the dicalcium silicate phase (β-Ca_2_SiO_4_), as confirmed by XRD.^57^ Additionally, the SEM analysis exhibits the presence of the particles in the form of a glassy matrix, confirming the presence of the MgSiO_3_ phase.^58^ After co-precipitation (Fig. 8(b)), notable morphological changes are observed. Specifically, dense, sphere-shaped particles form on the sample's surface, indicating the presence of the whitlockite phase. The observed changes in the morphology related to the bioactive response of this bioceramic (WH-C2S) are ascribed to a sequence of reactions involving ion exchange, along with dissolution and precipitation processes.

**Fig. 9:**
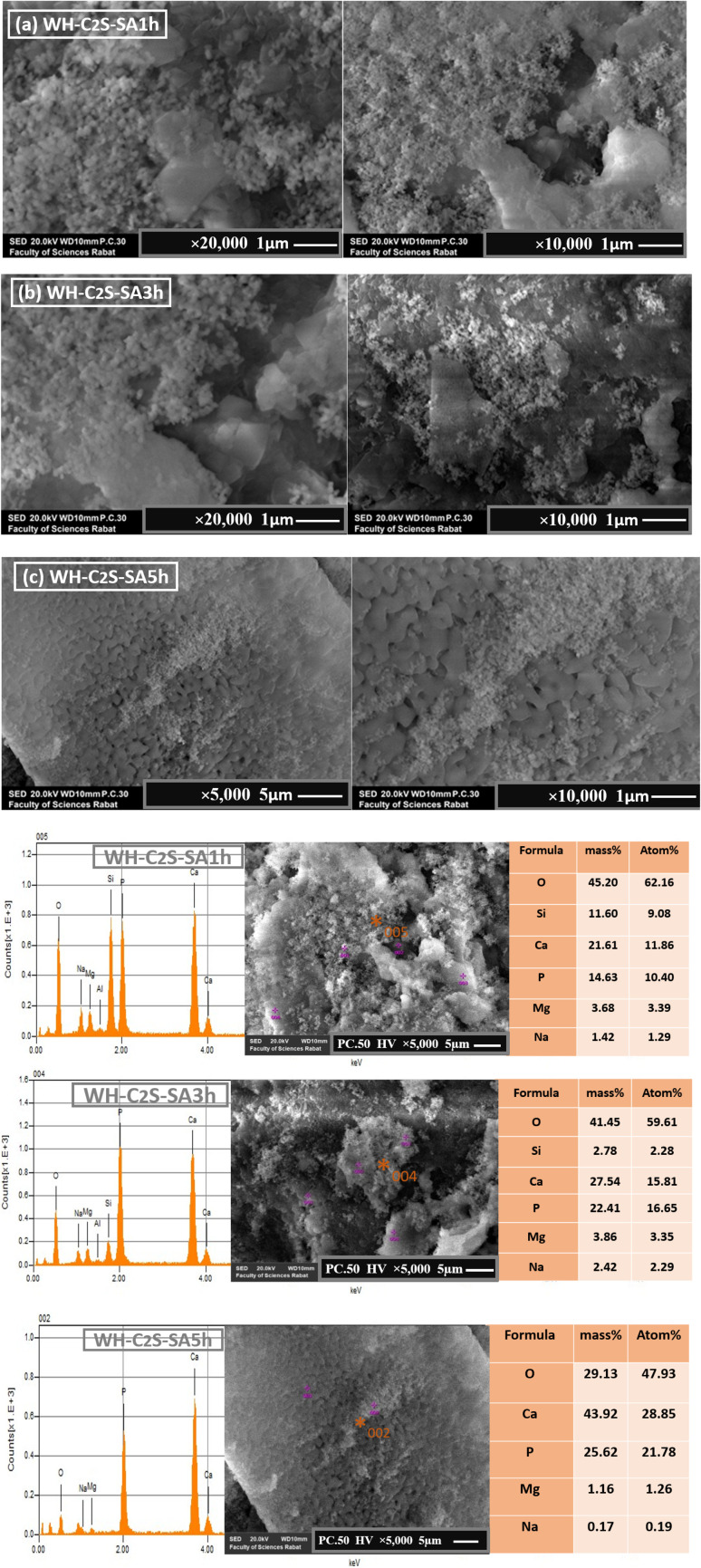
SEM images and EDS (×5 μm and ×1 μm) of the WH-C_2_S-SA1h (a), WH-C_2_S-SA3h (b), and WH-C_2_S-SA5h (c) samples after immersion in artificial saliva (SA).

**Fig. 10:**
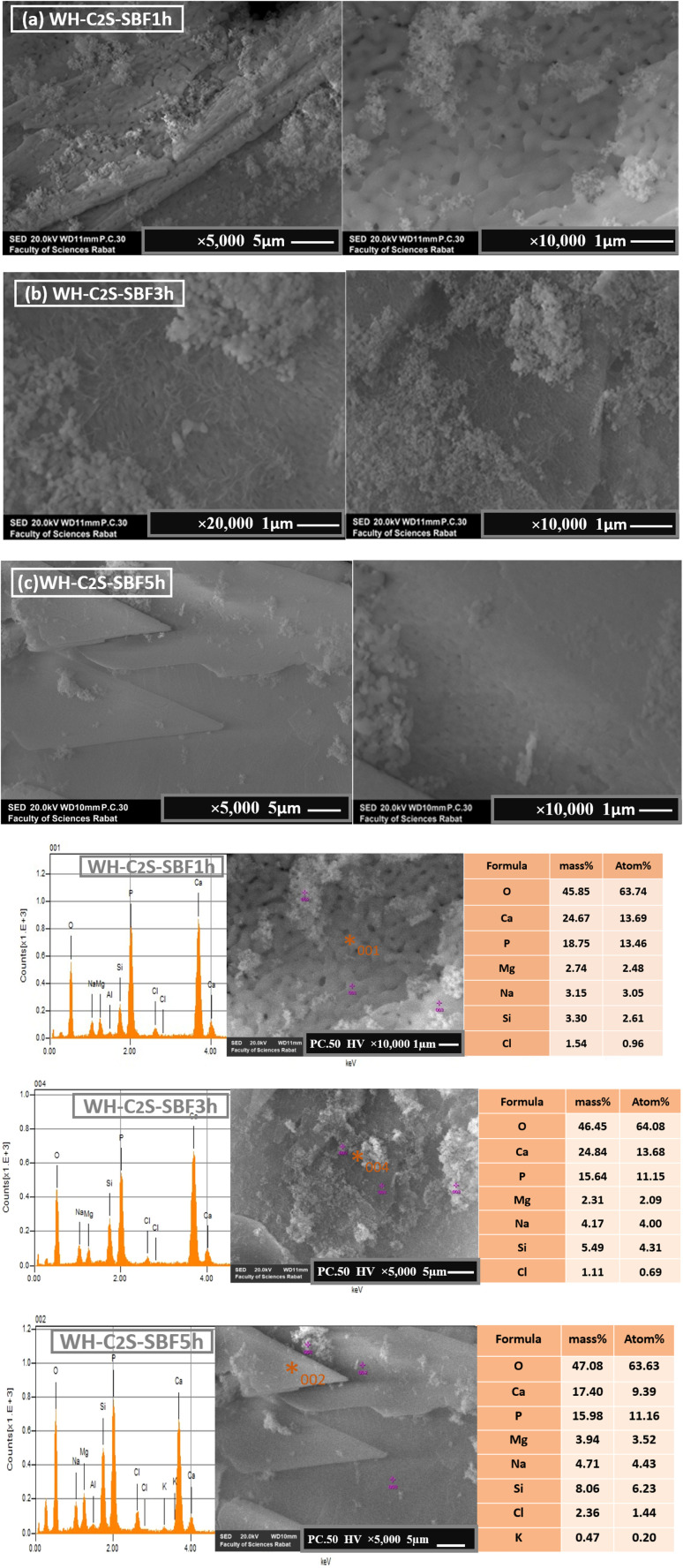
SEM images and EDS of the WH-C_2_S-SBF1h (a), WH-C_2_S-SBF3h (b), and WH-C_2_S-SBF5h (c) samples after immersion in the simulated body fluid (SBF) ×5 μm and ×1 μm.

A particle-size analysis was carried out based on the SEM images (magnification ×10 000), revealing a distribution of grain sizes predominantly smaller than 0.1 μm, with an estimated average equivalent diameter of approximately 91 nm (Fig. 9 and 10). This fine particle size demonstrates the density of the material and the controlled particle growth. Porosity analysis by image thresholding made it possible to obtain a porosity rate of approximately 15.3%, indicating an overall compact microstructure but empty intergranular zones. Furthermore, we determined the average thickness of the formed particles (1 to 5 ± 0.3 μm) from five analyzed areas. The results demonstrate that with different immersion time intervals, the products exhibit significantly different morphologies. Specifically, after 1 hour of immersion in artificial saliva (Fig. 9(a) and 10(a)), a large number of dense, sphere-shaped particles were clearly observed on the plates.^59^ Subsequently, after 3 hours of immersion (Fig. 9(b) and 10(b)), these particles continued to grow and gradually covered the plates.^59^ Furthermore, after 5 hours of immersion, notable changes in surface morphology were observed, with the appearance of interconnected sinuous paths.^60^ In addition, the morphologies of the WH material underwent significant changes following its immersion in a simulated body fluid solution. As illustrated in Figures (Fig. 9(c) and 10(c)), the spherical crystals developed an interconnected network structure.^61^ Notably, after 1 hour in the simulated body fluid, the grains had grown significantly and were well joined to each other, with notable consolidation achieved after 5 hours.^62^ This consolidation is attributable to the immersion duration, which directly influences the formation of the calcium phosphate layer on the surface of the bioceramic.^62^ The change in the morphological results of the bioactive response of our bioceramic (WH-C_2_S) could be attributed to a series of reactions involving ion exchanges, as well as dissolution and precipitation processes. Due to the rapid ion exchange between the bioactive solution and the bioceramic, which accelerates along with the hydration reaction, the sample dissolves rapidly when in contact with the bioactive solution. During this hydration, the concentration of hydroxide ions (OH^−^) increases. As a result, the conversion of hydrogen phosphate ions (HPO_4_^2−^) into phosphate (PO_4_^3−^) ions is facilitated by the increased OH^−^ concentration.^61^ Additionally, the silicate species in the bioactive solution, mainly in the form of Si(OH)_4_, react with the bioceramic surface to form silanol groups (Si–OH), which facilitate the formation of a silica layer.^63^ As a nucleation agent, this silica layer stimulates the calcium phosphate layer's subsequent deposition on the bioceramic. Moreover, the bioceramic's (WH-C_2_S) surface has a negative charge due to the OH^−^ and PO_4_^3−^ ions, and thus, it attracts calcium ions (Ca^2+^) from the bioactive solution. As a result, the surface acquires a positive charge, which promotes and enhances the growth of the whitlockite phase layer.^64^ Moreover, the bioactive solution's calcium (Ca^2+^) and magnesium (Mg^2+^) ions provide a second source of calcium and magnesium for the WH growth. When the ion concentration in the bioactive solution reaches its limit, crystalline nucleation develops, followed by crystalline growth.^65^ Moreover, the Mg^2+^ concentration from the bioactive solution appears to improve the microstructure and microhardness of the samples.^66^ Additionally, the higher concentrations of Ca^2+^ and Mg^2+^ in the simulated body fluid compared to those in the artificial saliva promote the predominant growth of the whitlockite phase in the SBF.^65^ Furthermore, the WH phase exhibits a good biological response in the simulated body.^66^

## 4. Conclusion

In this study, bioceramic whitlockite was synthesized from dicalcium silicate using limestone powder as the source of CaCO_3_ and soda-lime glass powder as the source of SiO_2_. The results of this study can be summarized into the following major conclusions:

- The bioceramic whitlockite exhibited good bioactivity in both saliva (SA) and simulated body fluid (SBF).

- The formation of the hydroxyapatite phase was observed after 5 hours of immersion in both artificial saliva and simulated body fluid.

- The predominant presence of the whitlockite phase in SBF compared to the case in saliva is attributed to its higher percentage in human blood compared to its percentage in natural saliva, making it an important component of the human bone structure.

- The micrographs obtained by SEM and EDS analysis show that after 1 hour in the simulated body fluid, the grains had grown significantly and were well joined to each other, with notable consolidation achieved after 5 hours.

- The whitlockite phase exhibited a good bioactivity response in the simulated body fluid.

- This study is based on the valorization of industrial by-products, such as limestone dust and soda-lime glass, as raw materials, thus proposing a methodological approach that is both sustainable and economical for the synthesis of bioceramics.

- The findings indicate that whitlockite-dicalcium silicate (WH-C_2_S) bioceramics are suitable for applications in bone regeneration and repair.

- The future studies will explore biological tests, such as cytotoxicity and cell viability assessments, to validate the biomedical potential of the synthesized material.

## Conflicts of interest

There are not conflicts to declare.

## Data Availability

No specific additional data are available for this study. All cited data can be found in the manuscript and the supplementary files.
